# Clustering of leprosy beyond the household level in a highly endemic setting on the Comoros, an observational study

**DOI:** 10.1186/s12879-019-4116-y

**Published:** 2019-06-07

**Authors:** Nimer Ortuno-Gutierrez, Abdallah Baco, Sofie Braet, Assoumani Younoussa, Aboubacar Mzembaba, Zahara Salim, Mohamed Amidy, Saverio Grillone, Bouke C. de Jong, Jan Hendrik Richardus, Epco Hasker

**Affiliations:** 1 Projects Department, Damien Foundation, Boulevard Leopold II, 263, PO B-1081, Brussels, Belgium; 2 National Tuberculosis and Leprosy control Program, Moroni, The Union of, Comoros; 3 Institute of Tropical Medicine, Antwerp, Belgium; 4 Erasmus MC, University Medical Center Rotterdam, Rotterdam, The Netherlands

**Keywords:** Mapping, Clustering, Leprosy, Post-exposure prophylaxis, Transmission, Single dose rifampicin

## Abstract

**Background:**

The island of Anjouan (Comoros) is highly endemic for leprosy with an annual incidence of 5–10/10,000. In May/June, 2015 single-dose Rifampicin post-exposure prophylaxis (SDR-PEP) was administered to 269 close contacts of 70 leprosy-patients in four villages as a pilot programmatic intervention. Two years later we revisited the villages for follow-up investigations. The main aim of our study was to quantify spatial associations between reported leprosy cases before and after PEP implementation. A secondary aim was to assess the effect of this single round of SDR-PEP at the individual level.

**Methods:**

We conducted door-to-door leprosy screening in all four villages in August/September, 2017. We screened all consenting individuals for leprosy and recorded geographic coordinates of their household. We also recorded whether they had received SDR-PEP and whether they had been diagnosed with leprosy, before or after the 2015 intervention. We fitted a Poisson model with leprosy as outcome and distance to the nearest pre-intervention case and SDR-PEP as predictors.

**Results:**

During the survey we found 114 new cases among 5760 contacts screened (2.0% prevalence), in addition to the 39 cases detected in the two preceding years. We found statistically significant associations of incident leprosy with physical distance to index cases ranging from 2.4 (95% confidence interval (95% CI) 1.5–3.6) for household contacts to 1.8 (95% CI 1.3–2.5) for those living at 1–25 m, compared to individuals living at ≥75 m.

The effect of SDR-PEP appeared protective but did not reach statistical significance due to the low numbers, with an incidence rate ratio (IRR) of 0.6 (95% CI 0.3–1.2) overall, and 0.5 (95% CI 0.2–1.3) when considering only household contacts.

**Conclusions:**

This pilot demonstrated an increased risk of leprosy in contacts beyond the household, therefore a wider circle should be considered for chemoprophylaxis. Baseline surveys and extended contact definitions are essential for improving SDR-PEP effectiveness.

## Background

Leprosy is an infectious disease caused by *Mycobacterium leprae* [1]. *M. leprae* is transmitted through the air [2] and after an incubation period of several months to 20 years, provokes skin lesions and nerve damage. Prolonged delay in diagnosis and treatment may cause permanent disability [3], which often leads to social stigma [4].

In 1991, the 44th World Health Assembly (WHA) set the year 2000 as a target to eliminate leprosy as public health problem, defined as a global prevalence of less than one leprosy case per 10,000 population [5]. Early diagnosis and multidrug therapy (MDT) contributed to attaining this goal, together with changes in case definition, achieving a prevalence reduction from more than five million cases in the 1980s to less than 600,000 by the year 2000 [6]. Nevertheless, the number of new leprosy cases reported annually has remained above 210,000 since 2013 [7]. Combined with the persistence of leprosy in children, this implies that there is no decline of the transmission of *M. leprae*, a key step needed to achieve leprosy elimination.

The Global Leprosy Strategy 2016–2020 encourages implementation research on prevention of leprosy, including chemoprophylaxis [8, 9]. Single dose Rifampicin Post-Exposure Prophylaxis (SDR-PEP) given to the contacts of newly diagnosed leprosy cases has been documented as an effective strategy, reducing leprosy incidence at village/neighbourhood level by approximately 50–60% [10, 11]. The success of implementing SDR-PEP under programmatic conditions relies on the integration of passive detection, active case finding and a strong monitoring and evaluation system [12]. Learning from the experience with SDR-PEP implementation of leprosy control programs is key to help identify its optimal implementation modalities.

**Fig. 1:**
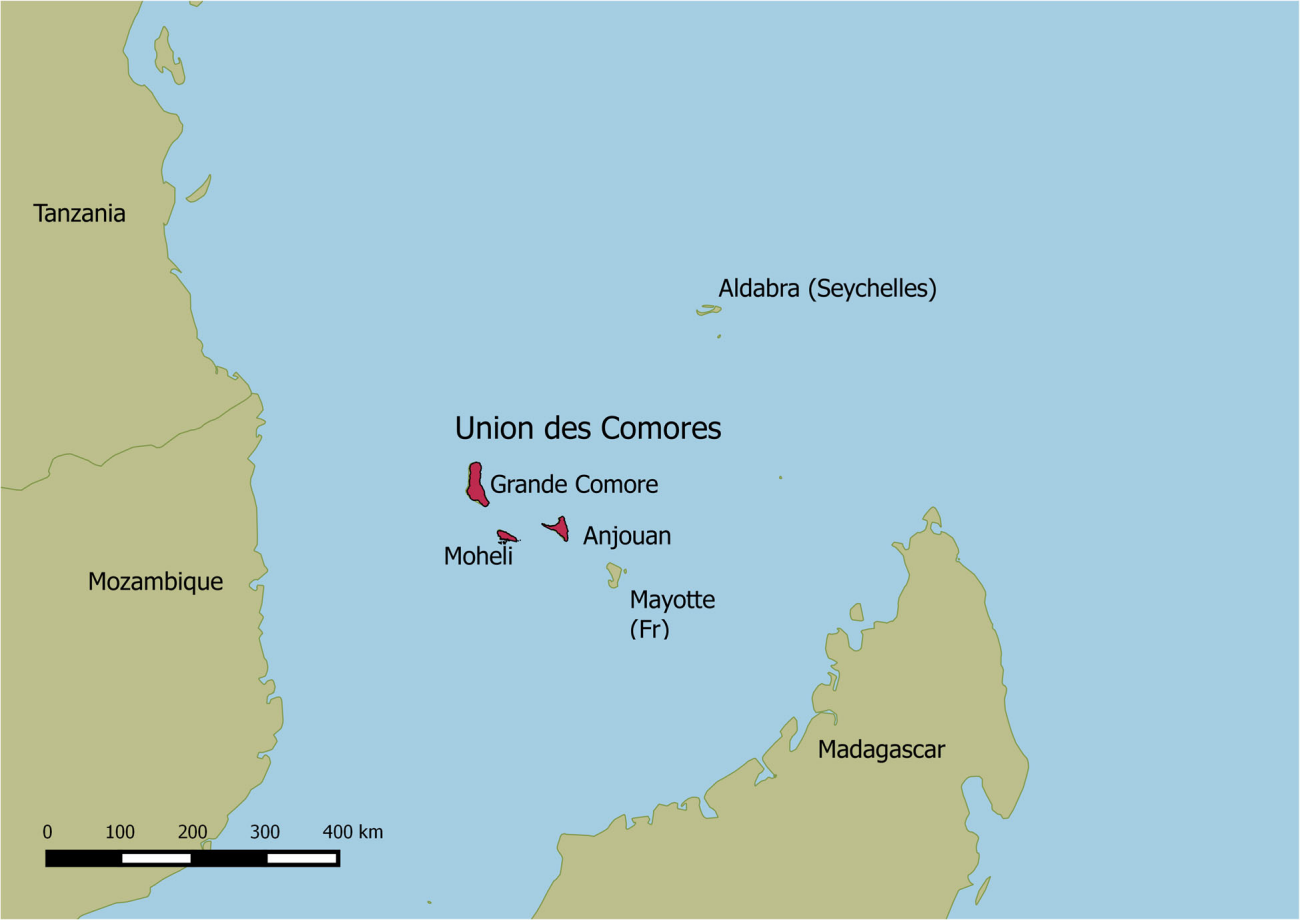
Overview map of the Union of Comoros. Map of Comoros and neighboring countries

The Comoros is an archipelago in the northern Mozambique Channel in the Indian Ocean. The closest neighbours are Tanzania (Northwest), Mozambique (West), Madagascar (South) and Seychelles (Northeast). Figure 1 shows an overview map of the Comoros. The country has approximately 810,000 inhabitants [13], distributed over three islands with distinct geological features. Of the total population, 51% live on the main island Grand Comore, 42% on Anjouan, where mountains limit the inhabitable land, and 7% on Mohéli. Leprosy has all but disappeared from Grand Comore since 1980, but persists on the two other islands [14].

Despite its modest population size, the Comoros is considered one of the 22 high leprosy burden countries [9]. In 2016, the national detection rate was 3.8/10,000 inhabitants. Out of 310 new leprosy cases detected, 83 (27%) were children (below 15 years of age) [7].

On the island of Anjouan, leprosy has been highly endemic for decades, with a reported incidence above 7/10,000 inhabitants, with more than 30% of new leprosy cases being children [15]. The leprosy control program on the Comoros was launched in 1978 and has since benefitted from the support of two international non-governmental organizations, Damien Foundation and AIFO (Associazione Italiana Amici di Raoul Follereau). Since 1986, tuberculosis (TB) and leprosy control have been integrated within the National Tuberculosis and Leprosy Programme (NTLP). On Anjouan 27 health facilities offer TB and leprosy care. The NTLP combines passive and active case finding to achieve early detection and cure. For active case finding, leprosy campaigns, where presumptive leprosy cases are examined in a designated location (also called ‘camp approach’), and contact tracing are in place [16]. These control strategies appear to have been effective in achieving early case detection, reflected in a proportion of new patients with visible disabilities of less than 2.5% [15]. The completion rate of leprosy treatment is also high; rates of above 85% for both multibacillary (MB) and paucibacillary (PB) leprosy have been reported for the period 2008 to 2014 [17]. Despite this apparently strong leprosy control program, the incidence of leprosy remains high.

In 2015, the NTLP decided to pilot implementation of SDR-PEP for household contacts in four highly endemic villages of Anjouan. One single round of SDR-PEP was provided to asymptomatic close contacts of recently diagnosed leprosy cases, with a focus on household contacts. The main objective of this intervention was to assess the feasibility of SDR-PEP under programmatic conditions and to document the lessons learnt before embarking on a larger prophylaxis strategy. The main aim of our study was to quantify spatial associations between reported leprosy cases before and after the 2015 intervention. The limited sample size precluded an accurate assessment of the effectiveness of SDR-PEP but we did take it into account as a potential confounder.

## Methods

### Setting

The study took place on Anjouan, the second largest island of the Comoros, with approximately 340,000 inhabitants. Anjouan has eight administrative districts, totalling 93 villages. Case finding for leprosy on Anjouan is based on a combination of active and passive approaches.

Four villages with an estimated population of 8400 had been selected for implementing SDR-PEP in May/June, 2015. A total of 269 consenting close contacts from 70 households had received SDR-PEP, with rifampicin at routine dosing, around 10 mg/kg.

These persons were close contacts, mostly living in the same household as leprosy patients diagnosed over the preceding 3½-year period (since January 1st, 2012). In this period altogether 176 patients had been diagnosed in the four villages.

### Study design

The study was designed as a retrospective cohort study. During door-to-door surveys in August/September, 2017, all consenting individuals were examined clinically for leprosy. For each person examined we recorded whether or not the person had suffered from leprosy in the past, whether or not the person had received SDR-PEP in 2015, and whether or not the person was currently suffering from leprosy. For past leprosy patients we recorded the date of diagnosis. The diagnosis of leprosy was made on clinical criteria, following WHO guidelines [18], including examination for loss of sensation and nerve enlargement. In addition, skin biopsies were taken from newly diagnosed all patients identified during the survey. We also recorded geographic coordinates of all households visited.

### Data collection and mapping

All data, including geographical coordinates of households visited, were recorded using a custom designed Android application in ‘Open Data Kit’ (ODK). Data were triangulated with the register of new leprosy patients and their records on contacts that had received SDR-PEP. Geographic coordinates of all households visited were plotted on a map using the Quantum Geographic Information System (QGIS) software package, with indication of whether or not there had been leprosy cases in the 3½-year period pre-intervention (index cases), or in the 2-year period after the intervention (incident cases).

We then created for all households screened a variable indicating the distance to the nearest index-case household ranging from 0 m (same household), to 1–25, 26–50, 51–75, or more than 75 m. Thus, households were split into five categories of distance to an index-case.

### Statistical analyses

We fitted a Poisson model with the count of leprosy cases detected since July 2015 as dependent variable, and the log of the population examined as offset. As independent variables we assessed the five categories of physical distance to the nearest index case and having been provided SDR-PEP. Those living at more than 75 m were used as reference category. Village of residence was included by default to control for potential confounding by contextual factors.

We calculated incidence rate ratios (IRR) and their 95% confidence intervals (95% CI). To assess a potential interaction between SDR-PEP and physical distance to an index case, we recoded the distance variable to a binary variable set to ‘1’ for household contacts and ‘0’ for all others. Bearing in mind that SDR-PEP was primarily provided to household contacts, we did a separate analysis restricted to household contacts only. A *p*-value < 0.05 was considered statistically significant.

### Ethics

This study is part of a larger study for which ethical approval was obtained from the institutional review board of the Institute of Tropical Medicine and the ethics committee of the Antwerp university hospital (both in Belgium), as well as from the ethics committee on Anjouan (Comoros). All subjects provided verbal consent for screening which was carried out by the national leprosy control program as part of their active case finding strategy. Leprosy patients identified were enrolled in the main study (reported separately) after providing written informed consent. In case of illiterate individuals, a thumb print and a signature of an independent witness were sought. For minors below the age of 18 years, a parent or guardian provided informed consent.

## Results

During the surveys in 2017 we registered a population of 5908, out of which 5760, were screened for leprosy. Among those were 133 out of 176 former leprosy patients diagnosed in the 3½-year period before the intervention (January 2012 to May 2015) and 259 out of 269 close contacts who had been provided SDR-PEP in June, 2015. Out of those 259 close contacts, 240 (92.7%) were household contacts. At the time of the surveys we detected 114 new cases, equivalent to a prevalence rate of 198/10,000.

Thirty-nine more cases had been detected previously in the period since SDR-PEP was provided, resulting in a cumulative incidence of 153 new cases since June, 2015.

There were statistically significant associations with physical distance to the nearest index case, the IRR for household contacts being 2.4 times higher (95% CI 1.5–3.6) than for those living at more than 75 m. For non-household contacts living within 25 m of an index case there was still a statistically significant increase in risk (IRR 1.8, 95% CI 1.3–2.5), beyond 25 m associations became statistically non-significant (see Table 1). The interaction term between household contact and SDR-PEP was statistically not significant (*p* = 0.23).

**Table 1:** Frequency and risk of being diagnosed with leprosy in relation to having received SDR-PEP and physical distance (in meters) to the nearest index case in four villages of Anjouan (Comoros)

Factor	Population (*n* = 5760)	(%)	No. of leprosy cases (*n* = 153)	(%)	IRR	(95% CI)
SDR-PEP provided						
- Yes	259	(4.5)	7	(2.7)	0.6	(0.3–1.2)
- No	5501	(95.5)	146	(2.7)	Ref.	
Distance to index case						
- Same household	672	(11.7)	27	(4.0)	2.4	(1.5–3.6)
- 1-25 m	1373	(23.8)	49	(3.6)	1.8	(1.3–2.5)
- 26- 50 m	1604	(27.9)	36	(2.2)	1.2	(0.8–1.7)
- 51- 75 m	654	(11.3)	16	(2.4)	1.3	(0.8–2.1)
- > 75 m	1457	(25.3)	25	(1.7)	Ref.	

*SDR-PEP* = Single Dose Rifampicin – Post Exposure Prophylaxis, *Ref.* = reference category

Out of the 259 close contacts screened in 2017 who had received SDR-PEP in 2015, seven (2.7%) had developed leprosy versus 146 out of 5501 (2.7%) among those who had not received PEP. Controlling for distance to the nearest index case and village of residence, the IRR for SDR-PEP was 0.6 (95% CI 0.3–1.2).

When looking at household contacts only, the effect of PEP was stronger but still not statistically significant. Among 240 current household contacts that had received PEP, six cases had occurred (2.5%) versus 21 among 432 (4.9%) that had not received PEP. Controlling for village of residence, the incidence rate ratio was 0.5 but similarly not statistically significant (95% CI 0.2–1.3).

**Fig. 2:**
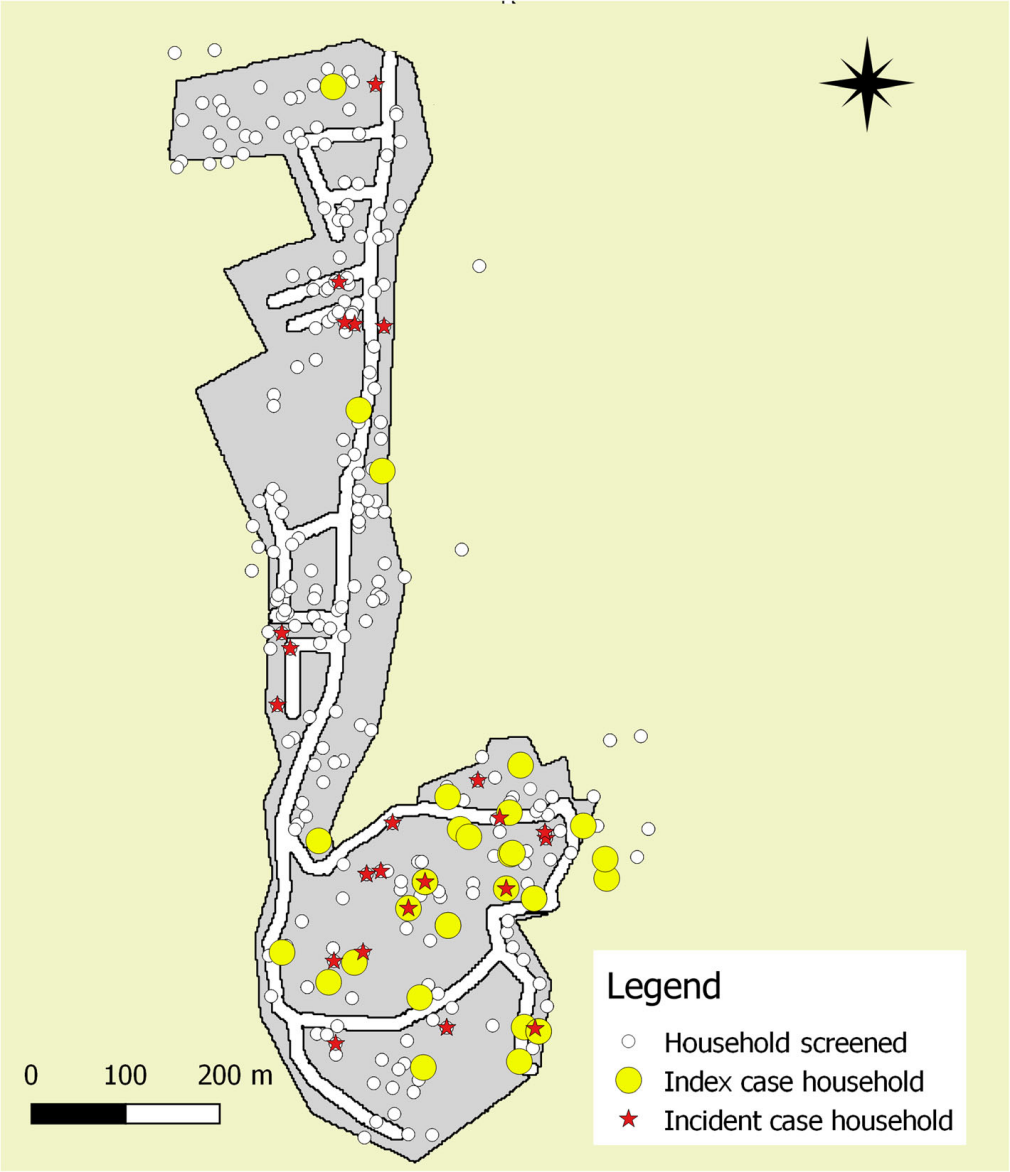
Distribution of index cases (January 2012–June 2015) and incident cases (July 2015–October 2017) in a village on Anjouan (Comoros). Legend: the map plots the household screened, incident leprosy cases households and incident leprosy cases in a village of Anjouan

Figure 2 shows the distribution of leprosy patients in one of the four villages. Despite a very high prevalence there was still apparent clustering, with incident cases clustered around index cases but also around other incident cases.

## Discussion

Through door-to-door surveys in four villages on the island of Anjouan of the Comoros that had been targeted for many years with active case finding activities using a camp approach (i.e. inviting people with skin conditions for free diagnostic screening in a central location in the villages), we found 114 new cases among 5760 contacts screened (2.0% prevalence). Thirty-nine more cases had been detected in the two preceding years. The chances of having leprosy were statistically significantly higher for those residing close to index cases (< 25 m). Two years earlier, in 2015, 269 close contacts from 70 households of leprosy patients in these villages had been provided with a single round of SDR-PEP.

We did assess the potential impact of the intervention and found that taking into account all contacts the overall effect of SDR-PEP appeared protective. But as we knew from the start, due to the low numbers (insufficient power) this effect was statistically not significant, with an incidence rate ratio (IRR) of 0.6 (representing a protective effect of 40%). When considering only household contacts the protective effect was somewhat stronger but still statistically not significant, with an incidence rate ratio of 0.5 (representing a protective effect of 50%). This is comparable with the results of the COLEP trial, which showed a 57% reduction in leprosy incidence over a two-year period after SDR-PEP was provided to contacts, in comparison to placebo [11]. Furthermore, 240 out of 259 contacts provided with PEP included in our survey (93%) were household contacts and therefore mainly blood-related. A higher effectiveness of SDR-PEP can be expected among non-blood related close contacts [19].

The main focus of our study was to assess the risk of being diagnosed with leprosy as a function of physical distance to an index case. Here we found a statistically significant IRR of 2.4 for household contacts compared to those living at more than 75 m distance. This result is close to the IRR of 2.1 (CI95% 1.6–2.7) for household contacts compared to non-contacts found in Malawi by Fine et al. [20], but lower than the IRR of 9.4 for household contacts compared to their neighbours found in Indonesia by Van Beers et al. [21]. The different grading of IRR could be explained by the type of population and their leprosy endemicity, however the physical distance to an index case related to the closeness and intensity of the contact is clearly the most important risk factor in our population, as also described elsewhere [22]. We found statically significant clustering up to 25 m from any index case.

This is similar to findings from Brazil by Moura et al. who reported equally high yields of active case finding among household contacts as among neighbours of index cases [23]. In our population the mix of different approaches of case finding could have weakened the spatial associations between index cases and incident cases detected because an important number of existing cases must have been missed at baseline.

In a trial reported by Bakker et al. [24] on highly endemic islands in Indonesia two doses of Rifampicin PEP were given three months apart, after which a threefold reduction in incidence of leprosy was observed on islands allocated to blanket treatment (i.e. treating the entire eligible population), whereas no effect was observed on islands where PEP was provided to household contacts and neighbours only. The islands in Indonesia had a high leprosy incidence (0.9% over three years in the non-intervention group), which is comparable to the incidence in our study villages on the Comoros (0.6% the last five years). With such high incidence levels, PEP given to close contacts alone may not have sufficient impact at the community level because there are many sources of transmission other than the reported cases. Such sources may include asymptomatically as well as symptomatically infected individuals [25, 26]. In such high prevalence situations, virtually all members of a community could be considered as a contact and the whole community would be eligible to a PEP intervention [27–29].

Whereas the Indonesian islands had only a few thousand inhabitants [24], Anjouan has more than 340,000. Subjecting them all to SDR-PEP seems not very feasible. If, on the other hand, it could be demonstrated that transmission clusters mostly within certain (parts of) high-endemic villages, targeting entire villages or parts of them would be feasible.

Two important lessons can be learned from this pilot study. Leprosy geographical clusters in space at the sub-village level, and targeting not only household members but also neighbours of index cases with active case finding and post exposure prophylaxis seems indicated. Secondly, in an environment with (very) high leprosy incidence, active case finding needs to be intensified prior to providing SDR-PEP to ensure that there is no hidden leprosy prevalence, otherwise many contacts of leprosy patients will not receive SDR-PEP.

## Conclusion

This pilot study demonstrated an increased risk of leprosy in contacts beyond the household, therefore a wider circle should be considered for chemoprophylaxis. Baseline surveys and extended contact definitions are essential for improving SDR-PEP effectiveness.

## Data Availability

The data supporting the findings of this publication are retained at the Institute of Tropical Medicine, Antwerp and will not be made openly accessible due to ethical and privacy concerns. Data can however be made available after approval of a motivated and written request to the Institute of Tropical Medicine at ITMresearchdataaccess@itg.be.

## Abbreviations

AIFO: Associazione Italiana Amici di Raoul Follereau
IRR: Incidence Rate Ratio
MB: Leprosy Multibacillary
MDT: Multidrug Therapy
NTLP: National Tuberculosis and Leprosy Programme
ODK: Open Data Kit
PB: Leprosy Paucibacillary
QGIS: Quantum Geographical Information System
SDR-PEP: Single-Dose Rifampicin Post-Exposure Prophylaxis
WHA: World Health Assembly

## References

[1] Hansen GHA. Undersøgelser Angående Spedalskhedens Årsager (investigations concerning the etiology of leprosy in Norwegian). Norsk Mag Laegervidenskaben. 1874(4):88.

[2] Araujo S, Freitas LO, Goulart LR, Goulart IM (2016). Molecular evidence for the aerial route of infection of Mycobacterium leprae and the role of asymptomatic carriers in the persistence of leprosy. Clin Infect Dis.

[3] Yawalkar SJ, Yawalkar SJ (2009). Deformities and their management. Leprosy for medical practitioners and paramedical workers. Basle, Switzerland: Novartis Foundation for Sustainable Development.

[4] Grzybowski A, Sak J, Pawlikowski J, Nita M (2016). Leprosy: social implications from antiquity to the present. Clin Dermatol.

[5] World Health Assembly. World Health Assembly 44. Resolutions and decisions. Leprosy: World Health Organization 1991 [Available from: http://www.who.int/neglected_diseases/mediacentre/WHA_44.9_Eng.pdf?ua=1.

[6] World Health Organization. Global leprosy situation 2000, vol. 4. Geneva; 2002. JANUARY 2002. Report No.: 35.

[7] World Health Organization. Global leprosy update, 2016: accelerating reduction of disease burden. Geneva; 2017 SEPTEMBER 2017. Report no. p. 35.

[8] World Health Organization. Global Leprosy Strategy 2016–2020. Accelerating towards a leprosy-free world. India: WHO Library Catologuing-in-Publication data. 2016:34.

[9] World Health Organization (2016). Global leprosy strategy 2016–2020. Accelerating towards a leprosy-free world. Operational manual. India: WHO library cataloguing-in-publication data.

[10] Ferreira SMB, Yonekura T, Ignotti E, Oliveira LB, Takahashi J, Soares CB (2017). Effectiveness of rifampicin chemoprophylaxis in preventing leprosy in patient contacts: a systematic review of quantitative and qualitative evidence. JBI Database System Rev Implement Rep.

[11] Moet FJ, Oskam L, Faber R, Pahan D, Richardus JH (2004). A study on transmission and a trial of chemoprophylaxis in contacts of leprosy patients: design, methodology and recruitment findings of. COLEP Leprosy Rev.

[12] Steinmann P, Reed SG, Mirza F, Hollingsworth TD, Richardus JH. Innovative tools and approaches to end the transmission of Mycobacterium leprae. Lancet Infect Dis. 2017. 10.1016/S1473-3099(17)30314-6 28693856

[13] United Nations Development Programme (UNDP). Human Development Report 2016. Human development for everyone. New York: United Nations Development Programme. 2016:286.

[14] Pattyn SR, Grillone S (1991). Leprosy in the Comores 1981-88. Ann Soc Belg Med Trop.

[15] Hasker E, Baco A, Younoussa A, Mzembaba A, Grillone S, Demeulenaere T (2017). Leprosy on Anjouan (Comoros): persistent hyperendemicity despite decades of solid control efforts. Leprosy review.

[16] Ministère de la Santé dlS, de la Cohésion Sociale et de la Promotion du Genre,. Plan National de Développement Sanitaire 2015–2019 de l'Union des Comores. Comores 2014. p. 84.

[17] Programme National de Lutte contre la Tuberculose et la Lèpre (PNTL). Rapport Annuel Lèpre 2016 - Union des Comores. Moroni; 2017.

[18] World Health Organization. Guide to eliminate leprosy as a public health problem. Geneva: WHO library cataloguing-in-publication data; 2000. p. 22.

[19] Schuring RP, Richardus JH, Pahan D, Oskam L (2009). Protective effect of the combination BCG vaccination and rifampicin prophylaxis in leprosy prevention. Vaccine..

[20] Fine PE, Sterne JA, Ponnighaus JM, Bliss L, Saui J, Chihana A (1997). Household and dwelling contact as risk factors for leprosy in northern Malawi. Am J Epidemiol.

[21] van Beers SM, Hatta M, Klatser PR (1999). Patient contact is the major determinant in incident leprosy: implications for future control. Int J Lepr Other Mycobact Dis.

[22] Moet FJ, Meima A, Oskam L, Richardus JH (2004). Risk factors for the development of clinical leprosy among contacts, and their relevance for targeted interventions. Lepr Rev.

[23] Moura ML, Dupnik KM, Sampaio GA, Nobrega PF, Jeronimo AK, do Nascimento-Filho JM (2013). Active surveillance of Hansen's disease (leprosy): importance for case finding among extra-domiciliary contacts. PLoS Negl Trop Dis.

[24] Bakker MI, Hatta M, Kwenang A, Van Benthem BH, Van Beers SM, Klatser PR (2005). Prevention of leprosy using rifampicin as chemoprophylaxis. Am J Trop Med Hyg.

[25] Bratschi MW, Steinmann P, Wickenden A, Gillis TP (2015). Current knowledge on Mycobacterium leprae transmission: a systematic literature review. Lepr Rev.

[26] Bakker MI, Hatta M, Kwenang A, Faber WR, van Beers SM, Klatser PR (2004). Population survey to determine risk factors for Mycobacterium leprae transmission and infection. Int J Epidemiol.

[27] Smith CM, Smith WC (2000). Chemoprophylaxis is effective in the prevention of leprosy in endemic countries: a systematic review and meta-analysis. MILEP2 study group. Mucosal Immunology of Leprosy The Journal of Infection.

[28] Richardus RA, van der Zwet K, van Hooij A, Wilson L, Oskam L, Faber R (2017). Longitudinal assessment of anti-PGL-I serology in contacts of leprosy patients in Bangladesh. PLoS Negl Trop Dis.

[29] van Hooij A, Tjon Kon Fat EM, Richardus R, van den Eeden SJ, Wilson L, de Dood CJ (2016). Quantitative lateral flow strip assays as user-friendly tools to detect biomarker profiles for leprosy. Sci Rep.

